# Therapeutic Effect, Rheological Properties and α-Amylase Resistance of a New Mixed Starch and Xanthan Gum Thickener on Four Different Phenotypes of Patients with Oropharyngeal Dysphagia

**DOI:** 10.3390/nu12061873

**Published:** 2020-06-23

**Authors:** Omar Ortega, Mireia Bolívar-Prados, Viridiana Arreola, Weslania Viviane Nascimento, Noemí Tomsen, Crispulo Gallegos, Edmundo Brito-de La Fuente, Pere Clavé

**Affiliations:** 1 Gastrointestinal Physiology Laboratory, CIBERehd CSdM-UAB, Hospital de Mataró, 08404 Mataró, Spain; oortega@csdm.cat (O.O.); mbolivar@csdm.cat (M.B.-P.); oarreola@csdm.cat (V.A.); wdo@csdm.cat (W.V.N.); ntomsen@csdm.cat (N.T.); 2 Centro de Investigación Biomédica en Red, Enfermedades Hepato-Digestivas (CIBERehd) Instituto de Salud Carlos III, 28029 Madrid, Spain; 3 Product and Process Engineering Center, Fresenius Kabi Deutschland GmbH, 61352 Bad Homburg, Germany; crispulo.gallegos-montes@fresenius-kabi.com (C.G.); edmundo.brito@fresenius-kabi.com (E.B.-d.L.F.)

**Keywords:** viscosity, rheology, amylase resistance, aspirations

## Abstract

Thickened fluids are a therapeutic strategy for oropharyngeal dysphagia (OD). However, its therapeutic effect among different phenotypes of OD patients has not yet been compared. We aimed to assess the therapeutic effect and α-amylase resistance of a mixed gum/starch thickener [Fresubin Clear Thickener^®^ (FCT)] on four phenotypes of OD patients: G1) 36 older; G2) 31 head/neck cancer (HNC); G3) 30 Parkinson’s disease; and G4) 31 chronic post-stroke. Therapeutic effect of FCT was assessed during videofluoroscopy using the Penetration-Aspiration Scale (PAS), for 5/20 mL boluses, at four levels of shear-viscosity (<50, 250, 1000 and 2000 mPa·s). The effect of α-amylase was assessed after 30 s of oral incubation. Patients had high prevalence of VFS signs of impaired efficacy (98.44%) and safety (70.31%) of swallow with a severe PAS score (4.44 ± 0.20). Most severe OD was in HNC (80.6% unsafe swallows). FCT showed a strong therapeutic effect on the safety of swallow at a range between 250–1000 mPa·s (74.19–96.67%, safe swallows in G1, G3, G4, and 58.06% in G2), without increasing pharyngeal residue. Viscosity was unaffected by α-amylase. Increasing shear-viscosity with FCT causes a strong viscosity-dependent therapeutic effect on the safety of swallow. This effect depends on the phenotype and is similar among older, Parkinson’s and post-stroke patients.

## 1. Introduction

Oropharyngeal dysphagia (OD) is a deglutition disorder, which has been classified in the last editions of the International Classification of Diseases ICD-9 and ICD-10 (787.2, R13) of the World Health Organization [1]. Prevalence is increasing with the aging of the population and it is a common condition among four main phenotypes of patients: older patients, patients treated for head and neck cancer and patients with neurological and neurodegenerative diseases [2]. OD has been recently recognized as a geriatric syndrome by two European Societies [3] and affects up to 27% of independently living and 51% of institutionalized older people [4,5] (1;2), 38.5% of patients with head and neck cancer [6], over 40% of post-stroke patients [7,8,9] and 82% in Parkinson’s disease [10]. OD causes two main groups of complications: impaired efficacy of swallow, which can lead to malnutrition and dehydration, and impaired safety of swallow with bolus penetrations into the laryngeal vestibule and tracheobronchial aspirations, leading to aspiration pneumonia and high mortality rates [11,12]. Videofluoroscopy (VFS) is the gold standard method to diagnose the biomechanical alterations of the oropharyngeal swallow response (OSR) [13]. It consists of a dynamic radiological exploration that evaluates the safety and efficacy of deglutition, characterizes the major signs of oropharyngeal dysfunction, quantifies the OSR and assesses the short-term effect of therapeutic strategies on patients with OD [13,14,15]. Delayed laryngeal vestibule (LV) closure has been recognized as the main mechanism of impaired airway protection in patients with OD leading to unsafe swallow [11].

Treatment of OD is currently mainly compensatory with fluid adaptation (volume and viscosity with thickening agents), texture-modified foods and the use of postures and maneuvers [16,17]. The use of thickening agents aims at maintaining the hydration status of patients with dysphagia, although evidence on the therapeutic effect of these compounds is weak [17]. The white paper published in 2016 by the European Society for Swallowing Disorders (ESSD) concluded that “there is evidence for increasing viscosity to reduce the risk of airway invasion and that it is a valid management strategy for OD. However, new thickening agents should be developed to avoid the negative effects of increasing viscosity on residue, palatability, and treatment compliance. New controlled trials should establish the optimal viscosity level for each phenotype of dysphagic patients and descriptors, terminology and viscosity measurements must be standardized” [16]. The thickeners’ composition is progressively changing from starches to gums. It is well known that standard starch-based thickeners greatly increase post-deglutitive oropharyngeal residue, especially in patients with deficient bolus propulsion such as older patients [11,16], increasing the risk of post-swallow aspirations [18]. Another disadvantage of starch-based thickeners is their poor acceptance by patients [19] causing a low compliance on prescriptions and treatments [20]. Moreover, in less than 30 s in the oral phase of swallow, the viscosity of starch-based thickeners can be dramatically reduced by oral salivary α-amylase as this breaks down starch molecules [21], reducing its therapeutic effect [22,23]. A new generation of thickening agents, such as xanthan gum or mixtures of gums and α-amylase-resistant modified starches, has shown better therapeutic properties for these patients [24,25,26,27] than the starch-based thickeners. The first generation of thickening agents marketed in EU was made of starch or starch derivatives (maltodextrins). These initial products offered a high protection but also a high oral and pharyngeal residue. New generation of thickening agents are composed by xanthan gum, which provide better properties on the safety, efficacy and a higher resistance to amylase. Differences on these groups of thickening agents are due to the mechanism of action: gum-based thickeners form hydrocolloids with water and remain stable on time, starch-based thickeners absorb water and swell, leading [26] to an inconstant viscosity behaviour on time and a strong affection of salivary amylase which hydrolyses the O-glycoside bonds of starch chains. Another important property that can affect safety of thickening agents is the shear-thinning behaviour, defined as a reduction of apparent viscosity under shear strain caused by bolus velocity in non-Newtonian fluids [28]. Bolus velocity through the gastrointestinal tract is maximal in the pharynx and can reach up to 30 cm/s [29]. It has been shown that bolus flow in the oral cavity occurs at shear rates of 50 s^−1^ approximately. However, during swallowing, the shear rate in the mesopharynx is over 300 s^−1^ and it can reach up to 900 s^−1^ in the hypopharynx [21,29]. 

Our aim was to assess and compare the therapeutic effect of a new thickening agent (Fresubin Clear Thickener^®^ [FCT], Fresenius Kabi), formulated with xanthan gum and modified starch, on the safety and efficacy of swallow plus kinematics of the swallow response. This involved four groups of patients with OD from several etiologies (aging, head and neck cancer, stroke and Parkinson’s disease). Moreover, we studied bolus flow resistance to α-salivary amylase and the shear-thinning behaviour of simulated boluses at three different levels of shear-viscosity (250 mPa·s, 1000 mPa·s and 2000 mPa·s, at 50 s^−1^).

## 2. Materials and Methods 

### 2.1. Study Design

**Figure 1:**
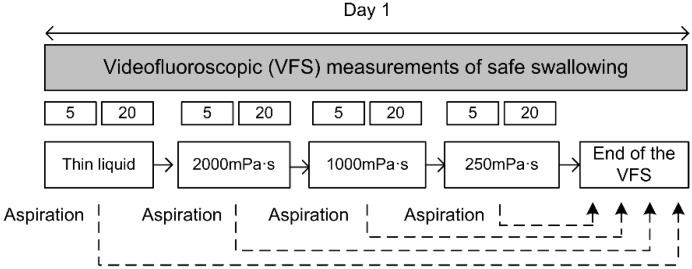
Study design.

This was a prospective, single-center clinical study on patients with OD caused by the four main etiologies (aging, head and neck cancer, stroke and Parkinson’s disease) to evaluate and compare the therapeutic effect and rheological characteristics of the thickening agent Fresubin Clear Thickener^®^ [FCT], (Fresenius Kabi Deutschland GmbH, Bad Homburg, Deutschland). First, patients were clinically assessed for OD with the Volume-Viscosity Swallowing Test (V-VST) [30] and if positive (with clinical signs of impaired efficacy and/or safety of swallow) they underwent VFS using four different viscosities (liquid <50, 250, 1000 and 2000 mPa·s) and 2 volumes (5 mL and 20 mL) for each viscosity to evaluate the therapeutic effect of the thickener and the kinematics of the swallow response. The general study design is presented in Figure 1. We also designed an in vitro study to assess the effect of α-amylase during the oral phase and the effect of shear-thinning during the oral and pharyngeal phase of this new thickener. 

The following data were also collected: sociodemographic characteristics of the population, functional capacity according to the Barthel Index [31], force with a hand dynamometer (Takei Analogue Dynamometer 5001, Takei Scientific Instruments, Japan), quality of life according to the EUROQoL-5D [32] and nutritional status according to body mass index (BMI) and the short form of the Mini Nutritional Assessment (MNAsf) [33]. 

### 2.2. Study Population

**Figure 2:**
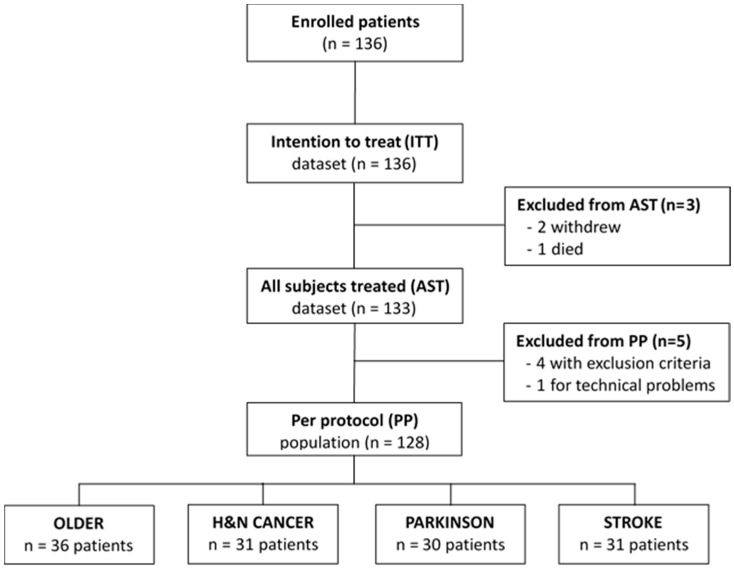
Study flow chart. Patients included in the study.

We studied 128 patients with OD divided in four groups according to OD etiology: a) 36 older patients, b) 31 patients following treatments for head and neck cancer (HNC), c) 31 post-stroke patients, and d) 30 Parkinson’s disease patients (Figure 2). Study population was prospectively recruited from the Gastrointestinal Physiology Unit of the Hospital de Mataró (Spain) between February 2016 and February 2018. Inclusion criteria were: patients over 18 years old, presenting OD according to the V-VST caused by any of the following etiologies: aging (>70 years), following treatment for head and neck cancer, stroke and Parkinson’s disease. Exclusion criteria were: pregnancy or lactation, unstable cardiopulmonary status, unstable medical conditions or major respiratory disease needing oxygen therapy. All of the participants were informed about the study and signed the informed consent form. Study protocol was approved by the ethical committee of the Hospital de Mataró with code 57/15 and was conducted according to the principles and rules laid down in the Declaration of Helsinki and its subsequent amendments and following the EU rules for clinical trials on humans (EU Clinical Trial Regulation (EU-CTR, EU No 536/2014)).

### 2.3. Study Product and Bolus Rheology

We used the Fresubin Clear Thickener^®^ (Fresenius-Kabi Deutschland GmbH, Bad Homburg, Deutschland) composed of xanthan gum, modified starch, maltodextrin, modified cellulose and flavouring. The solutions used for the in vivo study were prepared by adding the thickener to a given volume of water plus an X-ray contrast agent, and stirring thoroughly for 20 s as recommended by the manufacturer. Then, shear-viscosities (250 mPa·s, 1000 mPa·s; and 2000 mPa·s) were obtained by adding 0.7 g, 2.3 g, and 4.2 g, to 50 mL of liquid obtained by mixing 1:1 mineral still water and the X-ray contrast Omnipaque^®^ (GE Healthcare, La Florida, Spain) respectively, at room temperature (20 °C). For the in vitro rheological studies to assess the effect of salivary amylase and the effect of shear-thinning, boluses were prepared with 100 mL mineral still water and 1.75 g, 5.25 g, and 10.5 g respectively of FCT to achieve each level of viscosity. 

### 2.4. Swallowing Evaluation Measurements

#### 2.4.1. Screening (V-VST)

The V-VST was used to clinically screen patients for dysphagia. This test uses three volumes and three viscosities together with a pulse oximeter to assess clinical signs of OD. The procedure, algorithm and the psychometric characteristics of this tool have been described elsewhere [30,34].

#### 2.4.2. Instrumental Evaluation (Videofluoroscopy)

**Figure 3:**
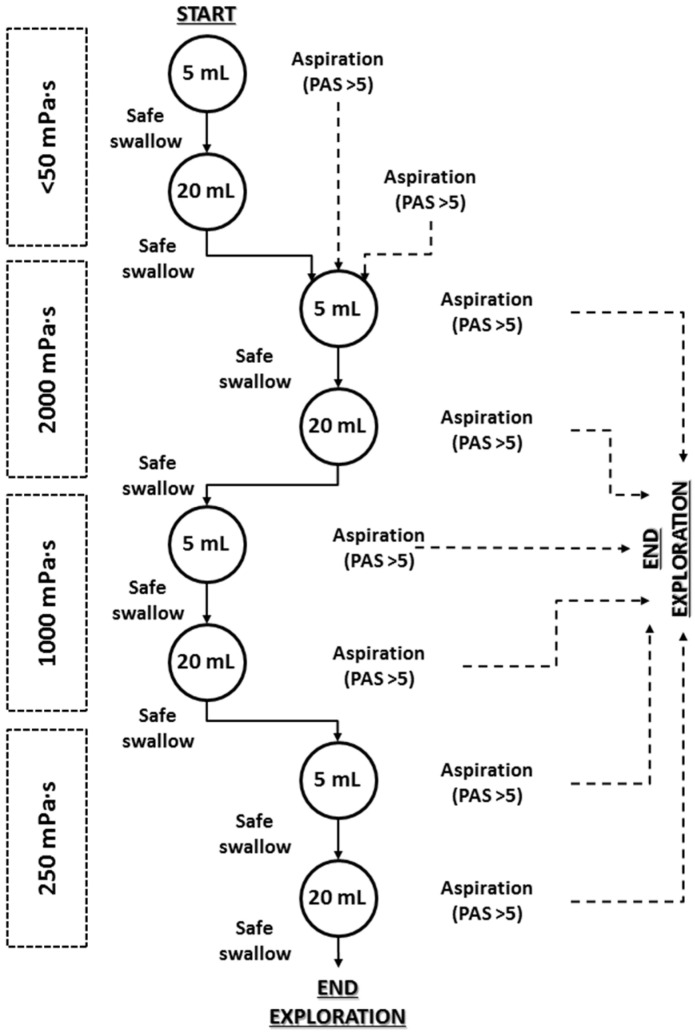
Study algorithm and safety stop rule for the videofluoroscopic exploration of the therapeutic effect of the different levels of viscosity. PAS: penetration-aspiration scale.

VFS was performed to assess the severity of OD, the metrics of the OSR and the therapeutic effect of the thickener. Patients were studied in lateral projection while seated and exploration included the oral cavity, pharynx, larynx, and cervical esophagus. Videofluoroscopic recordings were obtained with a Super XT-20 Toshiba Intensifier (Toshiba Medical Systems Europe, Zoetermeer, The Netherlands) and recorded at 25 frames/s using a Canon DM-XM2 E video camera (Canon Inc. Japan). The study algorithm was designed as an effort test to protect the patient from aspiration. It consisted of a series of 5 and 20 mL boluses of the viscosities selected. It began with thin liquid viscosity (<50 mPa·s) and continued with 2000 mPa·s followed by 1000 mPa·s and 250 mPa·s. As a safety rule for patients, if the patient presented an aspiration (PAS>5) at 5 mL thin liquid bolus, the 20 mL liquid bolus was skipped and the patient continued the exploration with the 5 mL 2000 mPa·s bolus [30]. If the patient presented an aspiration at 20 mL thin liquid, the exploration was continued with 5 mL 2000 mPa·s viscosity. Finally, if the patient presented an aspiration at any of the other boluses (after 20 mL thin liquid), the exploration was stopped as a safety rule to avoid further aspirations (Figure 3).

Videofluoroscopic signs of impaired safety and efficacy of deglutition were identified accordingly to previous studies [11,35]. Signs of impaired safety of swallow were assessed in each deglutition and included laryngeal vestibule penetrations and tracheobronchial aspirations, classified according to the PAS [36]. Unsafe swallow was defined as presenting a PAS score greater than two [36,37]. OSR was measured with the 5 mL bolus at each of the studied viscosities. Biomechanics of OSR were described as in previous studies [11,35], included the time to LVC, to upper esophageal sphincter opening (UESO), final kinetic energy (KE) of the bolus, bolus propulsion force, and mean bolus velocity. OSR was measured with specific software (Swallowing Observer; Image & Physiology SL, Barcelona, Spain) [25,27].

### 2.5. Rheological Characterization

We used a rotational viscometer (Thermo Fisher Scientific, Haake Viscotester^®^ 550, Germany) to analyze shear viscosity and shear-thinning behaviour at 25 °C, with shear rates from 1 to 1000 s^−1^. Viscosity values were measured at a shear rate of 50 s^−1^ and 300 s^−1^ respectively. An NV-rotor was used to analyze 250 mPa·s viscosity and an SV-DIN rotor to analyze higher viscosities (i.e., 1000 and 2000 mPa·s). RheoWin software, version 4.61 (Thermo Fisher Scientific, Waltham, MA, USA), was used for data processing. To assess the effect of salivary α-amylase during the oral phase of swallow, 15 mL boluses (250, 1000 and 2000 mPa·s) were given to the patients in a randomized order. Participants spit the bolus after 30 s of oral incubation and viscosities at both shear rates (50 and 300 s^−1^) were determined [29]. 

### 2.6. Palatability of the Product

Subjective palatability of the product was measured after each of the swallowed boluses at every volume and viscosity during VFS. Patients were asked to answer the following: What is your perception about the palatability of the given bolus? Each bolus was evaluated with a visual-analogue scale (VAS). The VAS scale was presented in a numerical form with values from 0 to 10 presented in a straight horizontal line of fixed length oriented from right to left. The descriptive term used for 0 was “bad” and for 10 was “excellent”. No descriptive terms were used for the rest of the numbers.

### 2.7. Adverse Events

Adverse events were reported and their relationship with the study product assessed from the initiation of any study procedure to the end of the study treatment follow-up according to the WHO and the Uppsala Monitoring Centre (WHO-UMC) category guideline [38]. 

### 2.8. Data and Statistical Analysis

Qualitative data are presented as relative and absolute frequencies and analyzed by the Fisher’s exact test (sex, VFS signs of impaired efficacy and safety of swallow) or the Chi-square test (MNA-sf categories, PAS score categories). The viscosity levels were compared with thin liquid by applying the McNemar’s test (VFS signs of impaired efficacy and safety of swallow between viscosities). Continuous data is presented as mean ± standard error of the mean (SEM) and compared using the *t*-test (intergroup comparisons) or paired *t*-test (intragroup comparisons); for those variables that did not follow a normal distribution, we used the nonparametric Mann–Whitney U test (intergroup comparisons), the Wilcoxon-paired test (intragroup comparisons) or the Kruskal–Wallis test for multiple comparisons with Dunn’s multiple comparison test. To assess normality, we used the D’Agostino and Pearson omnibus normality test. The main variable was prevalence of patients with safe swallowing at each one of the viscosities. These data were handled as binary by dividing the patients into two categories: a) patients who could swallow safely (PAS 1-2) vs. patients who could not swallow safely (PAS 3-8, including patients who discontinued the study due to the safety rule) over the ‘’per protocol’’ population. We compared prevalence of patients that could vs. could not swallow safely for each of the thickened viscosities compared with thin liquid. Safety of swallow of each patient for the whole VFS exploration or at a particular viscosity or level was expressed as the worst PAS score. Effect on penetrations (PAS 3-5) and aspirations (PAS 6-8) were also assessed. The efficacy of swallowing was also handled as binary data for oral and pharyngeal residue: if residue was observed in the oral cavity or at any of the three pharyngeal locations (pharyngeal wall, vallecular and pyriform sinus) the residue was present (yes), if no residue was observed at any of the locations the residue was absent (no). Results were interpreted according to the obtained *p*-value, the magnitude of the observed effect and their clinical and biological plausibility. Statistical significance was accepted if *p*-values were less than 0.05. Statistical analysis was performed with GraphPad Prism 6 (San Diego, CA, USA).

## 3. Results

### 3.1. Demographic Characteristics of the Population

We found many demographic differences among the study population groups (age, sex, functionality, BMI, force and health status self-perception) (Table 1). As a summary, older and stroke patients were the oldest and weakest (handgrip) groups, while those with HNC were the youngest and with the best functional capacity. In the older group, unlike the others, the majority of patients were women. Regarding nutritional status, MNA-sf evaluation showed high and similar percentages of malnourished or at risk of malnutrition patients (from 63.34% to 40%) in all groups. Patients with HNC had the lowest BMI. Finally, health status self-perception was quite low except for the HNC group (Table 1). 

### 3.2. Swallowing Evaluation by Videofluoroscopy (VFS)

#### 3.2.1. Videofluoroscopic Signs

(a) Effect of patient phenotype

VFS evaluation showed an overall study population with a high prevalence VFS signs of impaired safety (70.31%) and efficacy (98.44%) of swallow and residue and a severe PAS (4.44 ± 0.20). However, there was a different pattern of swallowing impairment depending on OD phenotype. Regarding impaired safety of swallow, we found a high prevalence of penetrations (46.67%–74.19%) and aspirations (13.33%–41.94%) in all groups and high mean PAS score (3.80–5.36) at thin liquid (<50 mPa·s). Patients with HNC had the most severe impairment of safety with a mean PAS score of 5.36 ± 0.41, 41.94% of them presenting aspirations and up to 25.81% of them, silent aspirations (Table 2). Older patients presented the highest prevalence of oral residue (91.67%) and those with stroke the lowest (61.29%, *p* < 0.01). HNC had the highest prevalence of pharyngeal residue (96.80%) while older patients had the lowest (66.67%, *p* < 0.01) (Table 2).

(b) Therapeutic effect of bolus viscosity with FCT

**Figure 4:**
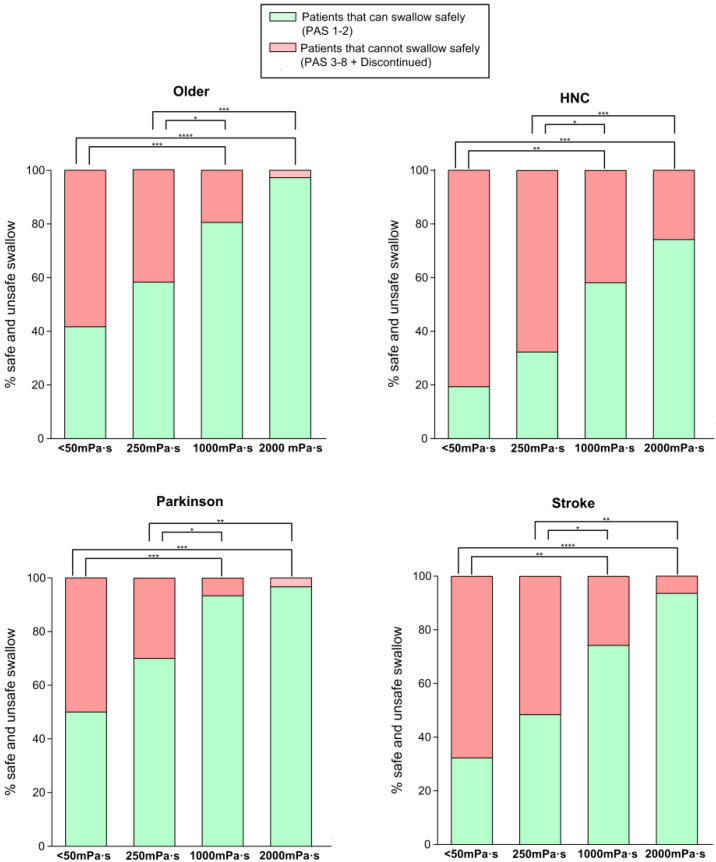
Percentage of patients who could swallow safely vs. those who could not swallow safely at each viscosity level. * *p*-value <0.05, ** < 0.01, *** <0.001, **** <0.001. HNC: head and neck cancer.

**Figure 5:**
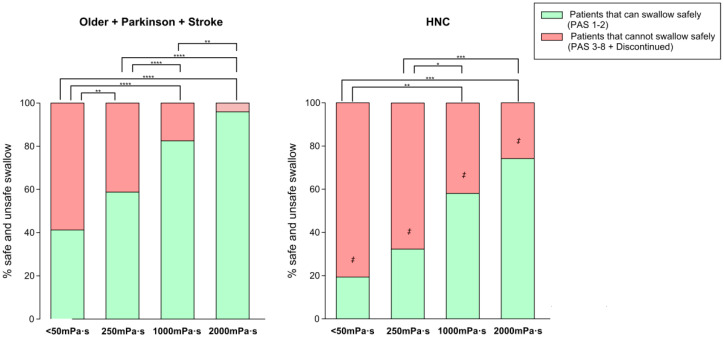
Percentage of patients who could swallow safely vs. those that cannot swallow safely comparing three groups together against HNC. Left: older, Parkinson’s and stroke patients; right: patients with head and neck cancer. * *p*-value <0.05, ** <0.01, *** <0.001, **** <0.001. ^‡^ *p*-value <0.05 Older + Parkinson’s + Stroke. HNC: Head and neck cancer.

**Figure 6:**
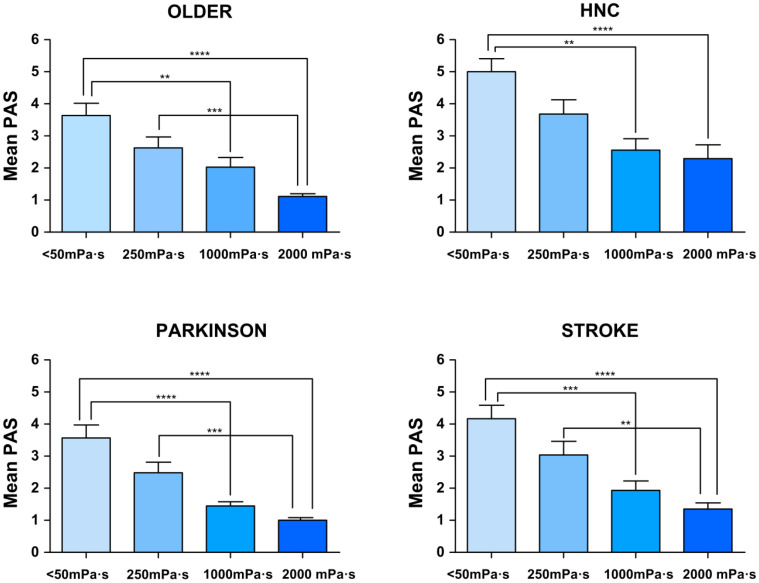
Mean penetration-aspiration scale (PAS) score in each viscosity of each of the study groups. * *p*-value <0.05, ** <0.01, *** <0.001, **** <0.001. HNC indicates head and neck cancer.

**Figure 7:**
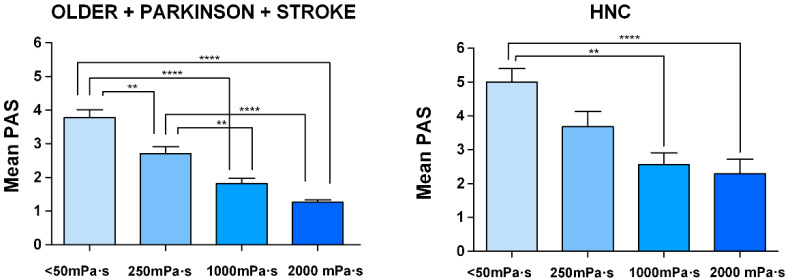
Mean penetration-aspiration scale (PAS) score in each viscosity comparing 3 groups together vs. HNC. Left: older, Parkinson’s and stroke patients; right: patients with head and neck cancer. * *p*-value <0.05, ** <0.01, *** <0.001, **** <0.001. HNC: Head and neck cancer.

Safety of swallow: We found a strong shear viscosity-dependent therapeutic effect on the safety of swallow, with a maximal significant therapeutic effect at 1000 mPa·s (80.56%, 96.67%, and 74.19% of safe swallows) for older, Parkinson’s and stroke patients, respectively. This therapeutic effect was greatly reduced in HNC patients, at 58.06%. (Figure 4 and Supplementary Figure S1). Further increase of viscosity up to 2000 mPa·s did not cause a significant increase in safety in any study group. We also found that the therapeutic effect of FCT depended on the phenotype of patient assessed: therapeutic effect was significantly reduced in HNC vs. all the other groups together (*p* < 0.05 <50 mPa·s; *p* < 0.01 250 mPa·s; *p* < 0.01 1000 mPa·s; and *p* < 0.001 2000 mPa·s) (Figure 5). However, it was similar between the three other groups, achieving 82.47% of patients with safe swallow at 1000 mPa·s, and 95.88% of patients with safe swallow at 2000 mPa·s (Figure 5). We also found an important reduction in the severity of OD measured with the PAS with significant changes when comparing <50 mPa·s vs. 1000 mPa·s and 2000 mPa·s in all groups of patients and 250 mPa·s vs. 2000 mPa·s in older, Parkinson’s and stroke groups, but not between 1000 and 2000 mPa.s (Figure 6), even when grouped together (Figure 7). The therapeutic range of FCT was defined between 250 and 1000 mPa·s. On the other hand, 250 mPa·s was selected as the minimum level of viscosity presenting a significant therapeutic effect (compared to thin liquid), and 1000 mPa·s as the maximal significant therapeutic effect compared to 2000 mPa·s.

**Figure 8:**
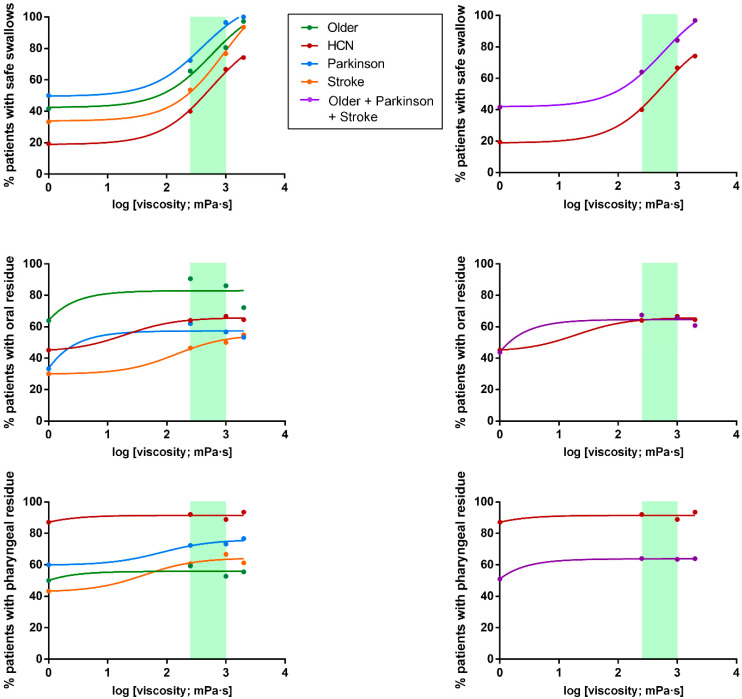
Viscosity-dependent effect of FCT on the safety and efficacy of swallow (oral and pharyngeal residue). Left: all groups of patients; right: older, Parkinson’s and stroke patients merged vs. patients with head and neck cancer. The therapeutic range has been marked with a green rectangle.

Efficacy of swallow: We found a significant increase in the prevalence of oral residue when we compared the study viscosities with thin liquid in all phenotypes (Supplementary Table S1). Older patients that had the highest prevalence of oral residue and also presented significant differences in oral residue when comparing 2000 mPa·s vs. 250 and 1000 mPa·s (Supplementary Table S1 and Figure 8). On the other hand, there were no differences regarding pharyngeal residue when we compared the tested viscosities in any of the studied phenotypes (Supplementary Table S2). The highest prevalence of pharyngeal residue was in HNC patients vs. the rest of the groups together: *p* < 0.001 <50 mPa·s; *p* < 0.01 250 mPa·s; *p* < 0.05 1000 mPa·s; and *p* = 0.068 2000 mPa·s in all the tested viscosities (Figure 8).

(c) Effect of bolus volume

We found no statistically significant differences in the prevalence of signs of impaired safety of swallow (PAS 3-8) when we compared 5 mL vs. 20 mL at any of the tested viscosities over the whole study population nor for each specific phenotype. However, we found statistically significant differences in all the viscosities when comparing the effect of volume on impaired efficacy of swallow (oral + pharyngeal residue), with a significant increase in residue at 20 mL (5 mL vs. 20 mL < 50 mPa·s: 61.90% vs. 90.38%, *p* < 0.0001; 250 mPa·s: 83.33% vs. 93.20%, *p* < 0.05; 1000 mPa·s: 87.70% vs. 97.37%, *p* < 0.01; and 2000 mPa·s: 81.10% vs. 95.16%, *p* < 0.001). Regarding the effect of FCT on the efficacy of swallow (oral + pharyngeal residue) for each specific phenotype, we found statistically significant differences when we compared 5 vs. 20 mL in <50 mPa·s in the older (*p* < 0.05), Parkinson’s (*p* < 0.01) and stroke groups (*p* < 0.01); and at 2000 mPa·s in the older group (*p* < 0.05). Taken together, our results show that increasing bolus volume from 5 mL to 20 mL has no impact on impaired safety of swallow but significantly increases oropharyngeal residue in all groups of patients together at all the tested viscosities.

(d) Dose-responses curves

FCT presented a strong therapeutic effect depending on the shear viscosity. The minimum viscosity tested (250 mPa·s) showed a significant therapeutic effect vs. thin liquid for all the phenotypes studied and it increased until the maximum viscosity assessed (2000 mPa·s) achieving >90% of safe swallows in older, Parkinson’s and stroke groups and 74.19% in HNC patients (Figure 8). However, no significant differences were seen between 1000 mPa·s and 2000 mPa·s for each group compared individually (80.56%, 96.67%, 74.19% and 58.06 of patients with safe swallow for each group at 1000 mPa·s, respectively). Accordingly, the threshold viscosity of safety was 250 mPa·s and maximal viscosity, 1000 mPa·s. Regarding dose-response curves for oral residue, we found a significant increase when comparing differences between <50 mPa·s and the rest of viscosities in all groups (Supplementary Table S1), but no differences between 250-2000 mPa·s and a higher prevalence of residue in the older group. Finally, the dose-response curve of pharyngeal residue was constant between viscosities for each phenotype but with a significant increase in prevalence in the HNC group compared to the rest of the study groups (Figure 8). 

#### 3.2.2. Oropharyngeal Swallow Response (OSR)

OSR at 5 mL liquid bolus was severely impaired in all studied groups when compared with our earlier studies on healthy volunteers (HV) [11]. Time to LVC was severely delayed ranging from 360 to 428 ms among the different groups (HV < 160 ms) and time to UESO was delayed and ranged from 240 to 281.33 ms (HV 200 ms). Regarding bolus kinematics, bolus propulsion force was weak (11.90 to 18.57 mN vs. HV 22 mN) leading to decreased bolus velocity from 0.24 to 0.33 m/s [11] (Table 3). Although patients with HNC presented the most delayed LVC (428.00 ± 35.11), there were no significant differences between groups. This impaired OSR, especially the severe delay in protection of the airway (LVC time), puts our patients at great risk of aspiration and residue through weak propulsion.

When we assessed the effect of viscosity on the OSR within groups, we found a reduction in the time to LVC in two groups of patients, older (*p* < 0.05, 2000 mPa·s vs. <50 mPa·s) and Parkinson’s 

(*p* < 0.05, 1000 mPa·s and 2000 mPa·s vs. <50 mPa·s). The other OSR parameters affecting oropharyngeal reconfiguration or bolus propulsion forces were not affected by increasing bolus viscosity with FCT (Table 3).

### 3.3. In Vitro Studies

(e) α-amylase effect and shear-thinning viscous behaviour

FCT presented strong resistance to salivary α-amylase effect. Viscosity was not affected by oral incubation at any of the viscosity levels tested compared with control samples without saliva for any phenotype assessed (Table 4). Surprisingly, we only found a slight but significant increase of viscosity in the 1000 mPa·s thickened fluid in patients with OD and stroke (Table 4; Supplementary Figure S2). FCT thickened fluids presented a non-Newtonian viscous behaviour (shear-thinning type) when submitted to shear. We found a similar percentage of viscosity reduction for all the samples between shear rates from 50 s^−1^ to 300 s^−1^, that was unaffected by salivary α-amylase in any group. Linear regression curves performed between a shear rate from 0 to 1000 s^−1^ are presented for each level of tested viscosity (Table 4). 

### 3.4. Palatability of the Product

Palatability was not significantly affected by increasing bolus viscosity. Palatability with the study product measured on a 10-point scale was similar in all the viscosities tested (6.73 ± 0.48 with liquid; 6.22 ± 0.48 with 250 mPa·s; 5.63 ± 0.43 with 1000 mPa·s; and 5.48 ± 0.42 with 2000 mPa·s). In addition, we did not find any difference in this item between volumes and viscosities in any of the study groups nor between groups (Supplementary Figure S3).

### 3.5. Adverse Events

No adverse events or serious adverse events related or not related to the study product were reported during the study period. 

## 4. Discussion

The main result of this study was that increasing bolus viscosity with FCT had a strong shear viscosity-dependent therapeutic effect by improving safety of swallow, with a threshold level of 250 mPa·s and maximal protection level at 1000 mPa·s^−1^. The therapeutic effect of FCT was very high and similar in older patients, patients with Parkinson’s disease and post-stroke patients compared with HNC patients, as FCT enabled safe swallow in 96% of patients of these three phenotypes. In contrast, the high severity of OD in HNC patients reduced this therapeutic effect at the same viscosity range. Finally, FCT is safe, well tolerated and not affected by salivary amylase in any of the study groups.

This study includes four of the most representative phenotypes of patients with OD: older, post-stroke, and patients with Parkinson’s disease and HNC. We have observed some similarities, but also very important differences, among these phenotypes of patients with OD. All of the patients presented high and similar prevalence of malnutrition, and a severe pattern of swallowing dysfunction with high prevalence of signs of impaired safety and efficacy of swallow and thus, were at great risk of developing further nutritional and/or respiratory complications [39]. Although HNC patients were the youngest with the best functionality and QOL, they had the most severe OD, with the highest prevalence of penetrations, aspirations, PAS and pharyngeal residue, and presented the lowest viscosity-dependent therapeutic effect. This is probably because HNC patients present OD as a consequence of structural changes secondary to the surgical process or radiochemotherapy (fibrosis, mucositis, etc.), in contrast to the other phenotypes that present OD mainly associated with impaired swallow physiology.

Older and stroke patients recruited in this study were demographically similar to those studied previously by our group [25,26,27,40]. Regarding the swallowing status of our phenotypes, older and stroke presented severe OD in terms of prevalence of signs of impaired safety (63.89% in older and 77.42% in stroke), impaired efficacy of swallow (100% in older and 96.67% in stroke), and PAS (mean PAS score of 4.08 ± 0.39 in older and 4.55 ± 0.41 in stroke). In addition, our patients also presented similar impaired OSR with a delayed time to LVC (336.25 ms in older and 390.67 ms in stroke) [11,40,41]. In a study by Vilardell et al. they found that time to LVC ≥340 ms predicted unsafe swallow in a group of chronic post-stroke patients with high diagnostic accuracy [40]. Patients from our study, including those with post-stroke OD, had a delayed time to LVC and thus a high prevalence of unsafe swallows. We also found that older patients presented the highest prevalence of oral residue. Oral residue is related to impaired bolus propulsion force due to weakness of pharyngeal muscles related to sarcopenia, a prevalent condition among older patients [42]. This specific phenotype conferred the lowest force measured with a handgrip dynamometer, but we did not find correlation between handgrip force and tongue bolus propulsion force in these patients. In contrast, the youngest and most functional patient group of the study, HNC patients, was that with the highest OD severity (highest prevalence of impaired safety of swallow and mean PAS score) related to the fact that they present severe pharyngeal anatomical alterations caused by surgery and concomitant treatments. In general, VFS signs for older, Parkinson’s and stroke were quite similar when comparing with those of the HNC phenotype and so the results for the three phenotypes were merged to discuss some results of the study vs. HNC values. Regarding the OSR, we only found a significant reduction in the time to LVC at 2000 mPa·s in older and Parkinson’s group. It is known now that older and stroke patients with OD present decreased pharyngeal sensitivity with impaired conduction and cortical integration of pharyngeal sensory inputs [43,44,45]. The reduction in the time to LVC at the higher viscosity could indicate that, at 2000 mPa·s, the bolus is more perceived in the pharynx increasing the sensory input and triggering a faster swallow response. We did not find any other effect of FCT in any other of the OSR parameters, showing that this therapy does not improve swallowing physiology but has a compensatory effect [11,16,25].

We observed that the therapeutic effect of increasing shear-viscosity with FCT is linked to the specific pathophysiology of OD in each phenotype, but it also presented a shear viscosity-dependent behaviour. We found a significant therapeutic effect with the lowest viscosity level assessed (250 mPa·s) as threshold viscosity. The percentage of patients with safe swallow increased for all groups until the highest viscosity level (2000 mPa·s), which presented high levels of protection. However, no significant differences were seen between 1000 mPa·s vs. 2000 mPa·s for each phenotype assessed individually. We also analyzed the differences between prevalence of aspirations regarding viscosity levels and we found no significant differences on the reduction of aspirations for any phenotype except for the older group and the number of aspirations increased for the highest viscosity in comparison to 1000 mPa·s even for the HNC phenotype. Differences that appeared for the older group at 2000 mPa·s were not clinically significant when compared with 1000 mPa·s when analyzing the percentage of safe swallows and the mean PAS value. Therefore, our results show a therapeutic range of 250–1000 mPa·s for FCT in the four phenotypes of patients. 

To complete the characterization of this thickener, we carried out a rheological analysis. FCT presented very good rheological properties, as it was resistant to α-salivary amylase, which demonstrated that viscosity remained constant in the oral cavity, increasing the therapeutic value of FCT compared with thickeners composed just by starch. The low adherence of patients to fluid modification is due to the low palatability of those products at high viscosity levels [46]. The basal palatability of this product was quite good and surprisingly, the palatability analysis performed showed no significant differences between liquid (<50 mPa·s) and the thickened viscosities (250-2000 mPa·s). These result shows that this product is well accepted by patients and it might present high adherence results among all phenotypes assessed in the present study. 

One of the limitations of the study comes from the screening (V-VST); although it offers a high sensitivity and specificity, some patients suffering from dysphagia can be missed. The main limitation of the study arose from the safety rule established during VFS algorithm, as some patients did not receive all the tested viscosities in order to avoid risks. This is an ethical decision that cannot be easily solved, as we are not able to push patients towards a risk of aspiration in order to respond to a research question. As previous studies have shown, there is higher risk of aspiration as we decrease the viscosity [24]. Another limitation is that we have used a palatability score for an acute situation, and that would not reflect the daily life acceptability of a patients that has to take this product in a chronic way. Thus, we believe that a longitudinal study should be done in order to know the exact palatability and acceptance of FCT in daily clinical practice.

We can conclude that the strong therapeutic effect of FCT is viscosity and phenotype-dependent and would work very well with those older, Parkinson’s and stroke patients, offering a therapeutic range of 250–1000 mPa·s and maximal protection with 1000 mPa·s. However, those patients with HNC, which present more severe anatomical and physiological alterations, showed a more reduced therapeutic effect of increasing shear-viscosity with FCT on the safety of swallow.

## 5. Conclusions

Increasing the bolus viscosity with Fresubin Clear Thickener^®^ had a strong viscosity-dependent therapeutic effect on patients with OD by improving the safety of swallow with a high protection level at 1000 mPa·s. The therapeutic range of Fresubin Clear Thickener^®^ in OD was established from 250 to 1000 mPa·s. This therapeutic effect is phenotype-dependent, the greatest therapeutic effect being in older, Parkinson’s and stroke patients. The high severity of OD in HNC patients reduces this therapeutic effect of increasing shear-viscosity at the same therapeutic range. In addition, Fresubin Clear Thickener^®^ is resistant to salivary α-amylase.

## Figures and Tables

**Table 1:** Demographic characteristics of the study population (continuous variables expressed as mean ± SEM).

	OLDER	HNC	PARKINSON	STROKE	*p*-Value
N	36	31	30	31	-
Age	82.96 ± 1.24	68. 29 ± 1.39 ****	72.34 ± 1.92 ****	79.42 ± 1.36 #### ŦŦŦ	<0.0001
Sex (female, %)	66.67 (24)	32.26 (10) **	20.00 (6) ***	35.48 (11) *	0.0008
Barthel (%) Optimum (100) (%) Sub-optimum (<100) (%)	78.33 ± 4.25 38.89 (14) 61.11 (22)	96.50 ± 1.68 * 67.34 (21) * 32.26 (10)	77.33 ± 4.48 # 23.33 (7) ### 76.67 (23)	74.83 ± 5.31#Ŧ 43.33 (13) 56.67 (17)	0.0007 0.005
MNA-sf (%) Well-nourished (12–14) At risk (8–11) Malnourished (0–7)	10.97 ± 0.38 47.22 (17) 44.44 (16) 8.33 (3)	11.50 ± 0.35 48.57 (17) 37.14 (13) 2.86 (1)	11.7 ± 0.47 # 60.00 (18) 33.33 (10) 6.67 (2)	10.6 ± 0.51## 36.67 (11) 56.67 (17) 6.67 (2)	0.326 0.610
BMI (kg/m^2^)	27.59 ± 0.88	23.97 ± 0.69 *	27.50 ± 0.85	27.78 ± 0.74	0.002
Handgrip Force (kg)	16.33 ± 1.42	22.78 ± 1.97	25.38 ± 1.78 **	17.77 ± 1.48Ŧ	0.0009
Health status self-perception (0–100)	63.57 ± 3.32	70.83 ± 3.85	56.25 ± 3.74#	58.67 ± 5.29	0.055

MNA-SF indicates mini nutritional assessment short form; BMC, body mass index; HNC, head and neck cancer. * *p*-value <0.05, ** <0.01, *** <0.001, ****<0.0001 vs. Older; # *p*-value <0.05, ## <0.01, ### <0.001, #### <0.0001 vs. HNC; Ŧ *p*-value < 0.05, ŦŦŦ <0.001 vs. Parkinson.

**Table 2:** Videofluroscopic signs of impaired efficacy and safety of swallow in the study groups (continuous variables expressed as mean ± SEM) for any viscosity level.

	ALL	OLDER	HNC	PARKINSON	STROKE	*p*-Value
N	128	36	31	30	31	
Impaired Efficacy (%) Oral Residue Pharyngeal Residue	98.44 (126) 82.81 (106) 80.47 (103)	100.00 (36) 91.67 (33) 66.67 (24)	100.00 (31) 80.65 (31) 96.80 (30) **	100.00 (30) 76.67 (23) ## 86.67 (26) ####	96.67 (29) 61.29 (19) ** ### 74.19 (23) # ^Ŧ^	nc 0.0002 <0.0001
Impaired Safety (%) Penetrations Aspirations Silent Aspirations (PAS = 8)	70.31 (90) 61.72 (79) 28.91 (37) 14.84 (19)	63.89 (23) 58.3 (21) 25.00 (9) 11.11 (4)	83.87 (26) 74.19 (23) 41.94 (13) 25.81 (8)	56.67 (17) # 46.67 (14) # 13.33 (4) # 13.33 (4)	77.42 (24) 67.74 (21) 35.48 (11) 9.67 (3)	0.076 0.135 0.071 0.256
Higher PAS score	4.44 ± 0.20	4.08 ± 0.39	5.36 ± 0.41	3.80 ± 0.40^#^	4.55 ± 0.41	0.038

PAS indicates penetration-aspiration scale; HNC, head and neck cancer; Impaired efficacy: fractional swallow, oral residue and pharyngeal residue. * *p*-value <0.05, ** <0.01 vs. Older; # *p*-value <0.05, ## <0.01, ### <0.001, #### <0.0001 vs. HNC; Ŧ *p*-value <0.05 vs. Parkinson; nc: not calculable.

**Table 3:** Comparison between viscosities of oropharyngeal swallow response among the study groups (continuous variables expressed as mean ± SEM).

		<50 mPa·s (5 mL)	250 mPa·s (5 mL)	1000 mPa·s (5 mL)	2000 mPa·s (5 mL)	*p*-Value
ALL	LVC (ms)	387.00 ± 13.32	359.00 ± 11.68	315.80 ± 9.59 *** #	316.20 ± 10.92 **** ##	<0.0001
	UESO (ms)	259.50 ± 9.79	257.20 ± 9.40	260.00 ± 8.61	290.10 ± 15.34	0.066
	KE (mJ)	0.96 ± 0.08	0.90 ± 0.06	0.83 ± 0.06	0.85 ± 0.10	0.112
	Force (mN)	14.87 ± 1.01	14.43 ± 1.08	13.19 ± 0.97	13.31 ± 1.42	0.228
	$\bar{x}$ bolus vel. (m/s)	0.26 ± 0.01	0.27 ± 0.01	0.27 ± 0.01	0.24 ± 0.01	0.230
OLDER	LVC (ms)	360.00 ± 20.85	336.25 ± 14.90	298.89 ± 14.78	284.57 ± 12.70 *	0.006
	UESO (ms)	260.00 ± 18.69	238.75 ± 13.85	232.22 ± 8.72	262.86 ± 14.21	0.304
	KE (mJ)	0.90 ± 0.11	0.90 ± 0.11	0.92 ± 0.10	0.80 ± 0.15	0.408
	Force (mN)	14.64 ± 1.63	15.24 ± 1.65	14.73 ± 1.23	12.48 ± 1.83	0.228
	$\bar{x}$ bolus vel. (m/s)	0.26 ± 0.02	0.27 ± 0.01	0.27 ± 0.01	0.25 ± 0.02	0.541
HNC	LVC (ms)	428.00 ± 35.11	370.00 ± 30.48	338.46 ± 26.37	360.00 ± 32.72	0.222
	UESO (ms)	240.00 ± 21.07	257.60 ± 19.47	280.00 ± 19.80	282.80 ± 25.26	0.153
	KE (mJ)	1.28 ± 0.25	0.82 ± 0.12	0.62 ± 0.07	0.81 ± 0.16	0.160
	Force (mN)	18.57 ± 3.08	12.13 ± 1.57	9.50 ± 1.11	12.28 ± 2.22	0.136
	$\bar{x}$ bolus vel. (m/s)	0.33 ± 0.03	0.28 ± 0.02	0.25 ± 0.01	0.27 ± 0.02	0.157
PARKINSON	LVC (ms)	373.33 ± 21.81	342.07 ± 26.68	293.33 ± 17.82 *	289.33 ± 12.08 *	0.009
	UESO (ms)	257.33 ± 20.18	249.66 ± 24.20	237.33 ± 16.49	241.33 ± 15.16	0.862
	KE (mJ)	0.89 ± 0.11	1.08 ± 0.18	0.99 ± 0.16	1.23 ± 0.32	0.963
	Force (mN)	14.18 ± 1.61	17.80 ± 3.30	16.50 ± 3.16	19.33 ± 4.78	0.946
	$\bar{x}$ bolus vel. (m/s)	0.27 ± 0.02	0.29 ± 0.02	0.29 ± 0.02	0.30 ± 0.03	0.920
STROKE	LVC (ms)	392.00 ± 27.55	390.67 ± 21.92	338.06 ± 18.35	335.48 ± 23.14	0.148
	UESO (ms)	281.33 ± 18.14	284.00 ± 16.98	296.77 ± 21.37	326.45 ± 23.18	0.421
	KE (mJ)	0.74 ± 0.07	0.77 ± 0.10	0.73 ± 0.10	0.58 ± 0.07	0.317
	Force (mN)	11.90 ± 1.14	12.17 ± 1.44	11.34 ± 1.47	9.26 ± 1.13	0.325
	$\bar{x}$ bolus vel. (m/s)	0.24 ± 0.01	0.24 ± 0.02	0.24 ± 0.02	0.21 ± 0.01	0.3367
OLDER + PARKINSON + STROKE	LVC (ms)	374.20 ± 13.47	356.40 ± 12.59	309.70 ± 9.88 *** #	302.5 ± 9.78 *** ##	<0.0001
	UESO (ms)	265.80 ± 11.00	256.40 ± 10.87	254.40 ± 9.51	276.70 ± 10.82	0.170
	KE (mJ)	0.85 ± 0.06	0.92 ± 0.08	0.88 ± 0.07	0.87 ± 0.12	0.220
	Force (mN)	13.66 ± 0.87	15.07 ± 1.31	14.22 ± 1.19	13.63 ± 1.72	0.147
	$\bar{x}$ bolus vel. (m/s)	0.26 ± 0.01	0.27 ± 0.01	0.27 ± 0.01	0.26 ± 0.01	0.267

$\bar{x}$ indicates mean; HNC, Head and neck cancer; PK, Parkinson; STR, Stroke; LVC, Laryngeal vestibule closure; UESO, upper esophageal sphincter opening; KE, kinetic energy; HNC, Head and neck cancer. * *p*-value <0.05, *** <0.001, **** <0.0001 vs. <50 mPa·s; # <0.05, ## <0.01 vs. 250 mPa·s.

**Table 4:** Viscosity percentage change between comparisons of control vs. samples after oral incubation (amylase effect) and shear thinning effect represented with the linear regression from 0 to 1000 s^−1^ shear rate. Positive values indicate increase and negative values decrease of viscosity.

	% Change (Viscosity at 50 s^−1^ before and after Oral Incubation)	*p*-Value	Shear-Thinning Effect (Linear Regression from 0 to 1000 s^−1^)	Correlation Coefficient‘r’
ALL	+7.85	0.637	fx = −0.91x + 4.18	0.99
	+7.16	0.135	fx= −0.93x + 4.71	0.99
	−2.96	0.560	fx= −0.91x + 4.94	0.99
OLDER	+30.47	0.062	fx= −0.83x + 4.067	0.99
	−4.01	0.471	fx= −0.83x + 4.39	0.99
	−2.29	0.659	fx= −0.81x + 4.69	0.99
HNC	+9.30	0.459	fx= −0.90x + 4.15	0.99
	+4.17	0.531	fx= −0.91x + 4.64	0.99
	−1.86	0.706	fx= −0.91x + 4.91	0.99
PARK.	+9.47	0.447	fx= −0.92x + 4.2	0.99
	+11.64	0.061	fx= −0.93x + 4.68	0.99
	−5.22	0.424	fx= −0.88x + 4.86	0.99
STROKE	−10.17	0.327	fx= −0.90x + 4.09	0.99
	+14.11	0.025	fx= −0.92x + 4.69	0.99
	−2.62	0.683	fx= −0.91x + 4.93	0.99

HNC: Head and neck cancer; PARK: Parkinson’s.

## Supplementary Materials

The following are available online at https://www.mdpi.com/2072-6643/12/6/1873/s1, Figure S1. Penetration Aspiration Scale (PAS) score frequency between viscosities and patients. PAS 1-2 indicates safe swallow and PAS 3-8, unsafe swallow; HNC, head and neck cancer. Figure S2. Effect of saliva (samples with saliva) on viscosity measured with a rotational viscometer within groups. Figure S3. Palatability of the study product presented for all patients valuated with a visual analogue scale (0-10). Table S1. Comparison between viscosities of oral residue among the study groups. HNC: Head and neck cancer. Table S2. Comparison between viscosities of pharyngeal residue among the study groups. HNC: Head and neck cancer.
