# Supplementary material for: Therapeutic Effect, Rheological Properties and α-Amylase Resistance of a New Mixed Starch and Xanthan Gum Thickener on Four Different Phenotypes of Patients with Oropharyngeal Dysphagia
Source: Nutrients. 2020 Jun 23;12(6):1873. doi: 10.3390/nu12061873 (PMC7353421; doi:10.3390/nu12061873)
Supplement: Supplementary file 1 [file nutrients-12-01873-s001.pdf]

SUPPLEMENTARY FIGURES

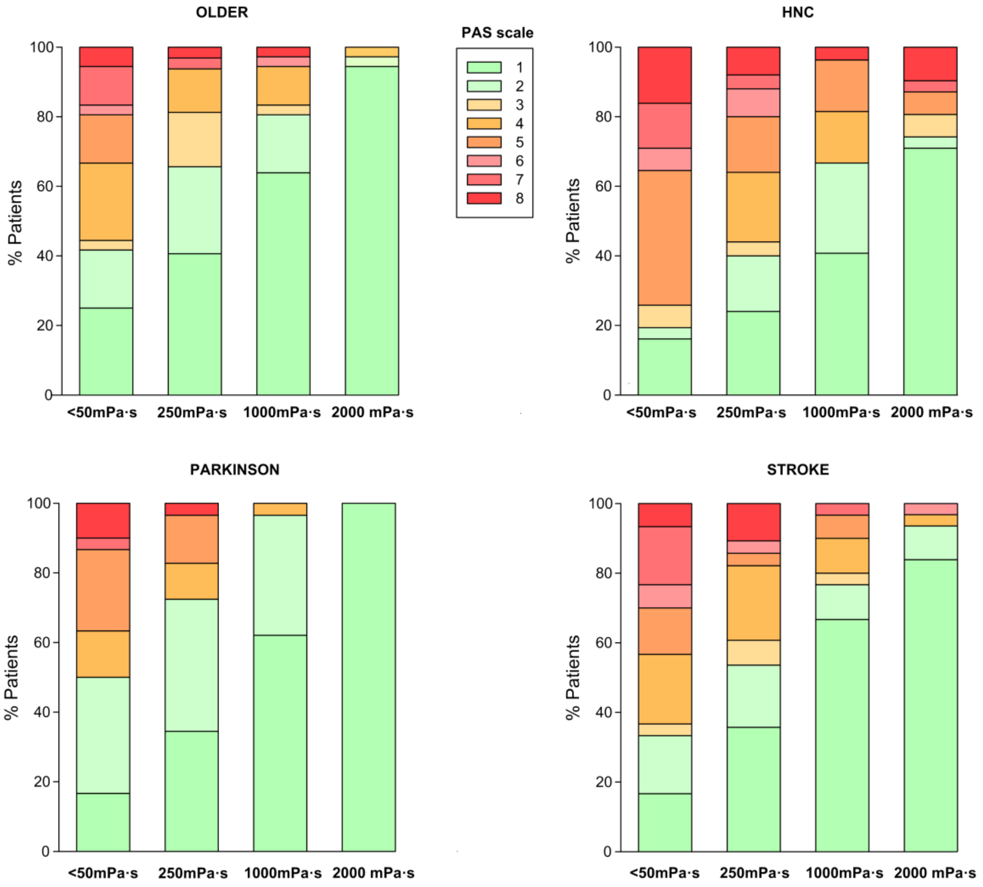

Figure S1. Penetration Aspiration Scale (PAS) score frequency between viscosities and patients. PAS 1–2 indicates safe swallow and PAS 3–8, unsafe swallow; HNC, head and neck cancer.

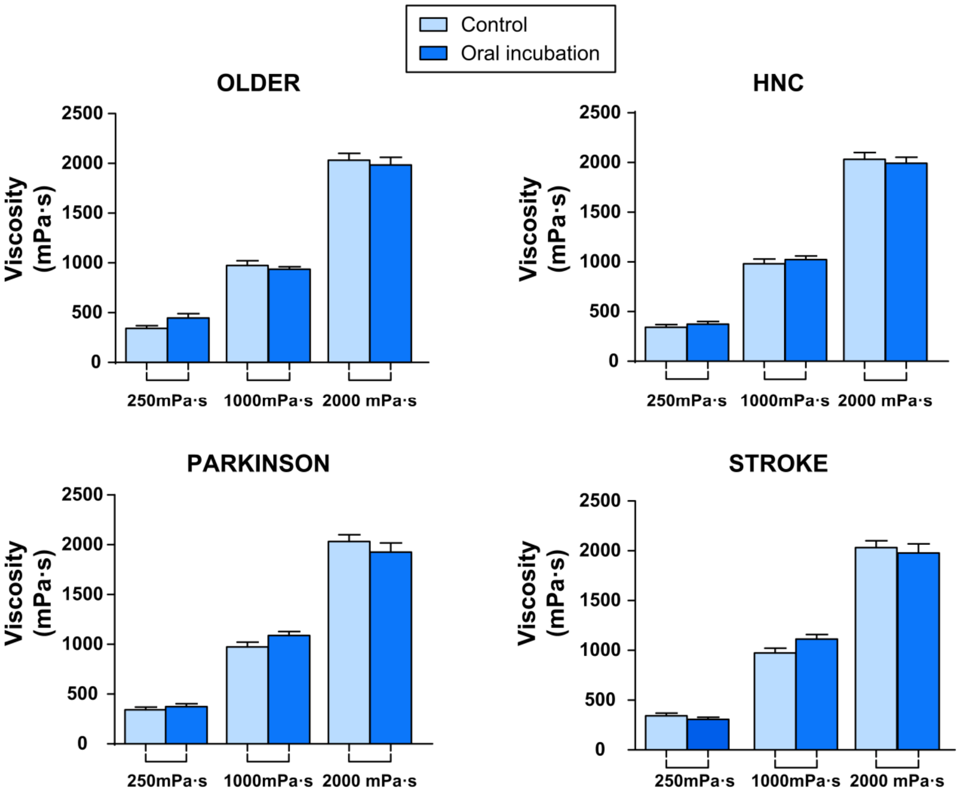

Figure S2. Effect of saliva (samples with saliva) on viscosity measured with a rotational viscometer within groups.

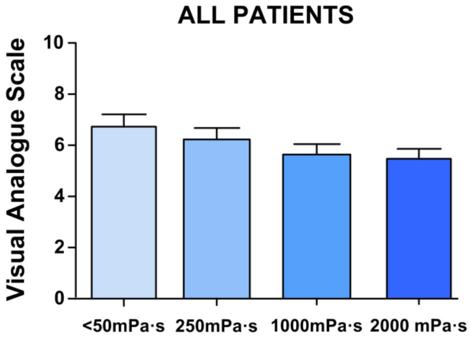

Figure S3. Palatability of the study product presented for all patients valuated with a visual analogue scale (0–10).

SUPPLEMENTARY TABLES

Table S1. Comparison between viscosities of oral residue among the study groups. *HNC: Head and neck cancer.*

**OLDER**	**<50mPa·s (n=36)**	**250mPa·s (n=32)**	**1000mPa·s (n=36)**	**2000mPa·s (n=36)**
**Oral residue**	63.89% (23)	90.63% (29)	86.11% (31)	72.22% (26)
<50mPa·s		0.008	0.013	0.752
250mPa·s			1.000	0.023
1000mPa·s				0.041

**HNC**	**<50mPa·s (n=31)**	**250mPa·s (n=25)**	**1000mPa·s (n=27)**	**2000mPa·s (n=31)**
**Oral residue**	45.16% (14)	64.00% (16)	66.67% (18)	64.52% (20)
Liquid		0.041	0.046	0.114
Nectar			0.683	0.617
Honey				0.683
Pudding				

**PARKINSON**	**<50mPa·s (n=30)**	**250mPa·s (n=29)**	**1000mPa·s (n=30)**	**2000mPa·s (n=30)**
**Oral residue**	33.33% (10)	62.07% (18)	56.67% (17)	53.33% (16)
<50mPa·s		0.027	0.070	0.077
250mPa·s			1.000	0.683
1000mPa·s				1.000

**STROKE**	**<50mPa·s (n=30)**	**250mPa·s (n=28)**	**1000mPa·s (n=30)**	**2000mPa·s (n=31)**
**Oral residue**	30.00% (9)	46.46% (13)	50.00% (15)	54.84% (17)
<50mPa·s		0.131	0.073	0.023
250mPa·s			0.683	0.683
1000mPa·s				0.617

Table S2. Comparison between viscosities of pharyngeal residue among the study groups. *HNC: Head and neck cancer.*

**OLDER**	**<50mPa·s (n=36)**	**250mPa·s (n=32)**	**1000mPa·s (n=36)**	**2000mPa·s (n=36)**
**Pharyngeal residue**	50.00% (18)	59.38% (19)	52.78% (19)	55.56% (20)
<50mPa·s		0.450	1.000	0.724
250mPa·s			0.683	1.000
1000mPa·s				1.000

**HNC**	**<50mPa·s (n=31)**	**250mPa·s (n=25)**	**1000mPa·s (n=27)**	**2000mPa·s (n=31)**
**Pharyngeal residue**	87.10% (27)	92.00% (23)	88.89% (24)	93.55% (29)
<50mPa·s		0.475	1.000	0.480
250mPa·s			1.000	0.480
1000mPa·s				1.000

**PARKINSON**	**<50mPa·s (n=30)**	**250mPa·s (n=29)**	**1000mPa·s (n=30)**	**2000mPa·s (n=30)**
**Pharyngeal residue**	60.00% (18)	72.41% (21)	73.33% (22)	76.67% (23)
<50mPa·s		0.371	0.221	0.074
250mPa·s			1.000	1.000
1000mPa·s				1.000

**STROKE**	**<50mPa·s (n=30)**	**250mPa·s (n=28)**	**1000mPa·s (n=30)**	**2000mPa·s (n=31)**
**Pharyngeal residue**	43.33% (13)	60.71% (17)	66.67% (20)	61.29% (19)
<50mPa·s		0.450	0.077	0.182
250mPa·s			0.371	1.000
1000mPa·s				1.000
